# Impact of the COVID-19 pandemic on perceived changes in responsibilities for adult caregivers who support children and youth in Ontario, Canada

**DOI:** 10.1192/bjo.2023.632

**Published:** 2024-01-24

**Authors:** Madeline Chiang, Roula Markoulakis, Anthony Levitt

**Affiliations:** Sunnybrook Research Institute, Toronto, Ontario, Canada; and College of Science, University of Notre Dame, South Bend, Indiana, USA; Sunnybrook Research Institute, Toronto, Ontario, Canada; and Department of Occupational Science and Occupational Therapy, University of Toronto, Ontario, Canada; Sunnybrook Research Institute, Toronto, Ontario, Canada; Family Navigation Project, Sunnybrook Research Institute, Toronto, Ontario, Canada; Hurvitz Brain Sciences Program, Sunnybrook Health Sciences Centre, Toronto, Ontario, Canada; and Department of Psychiatry, University of Toronto, Ontario, Canada

**Keywords:** Caregivers, caregiving responsibilities, children/youth, mental health, pandemic

## Abstract

**Background:**

The COVID-19 pandemic has created long-lasting changes in caregiving responsibilities, including but not limited to increased demands, loss of support, worsening mental and physical health, and increased financial worries. There is currently limited evidence regarding factors associated with perceived changes in caregiving responsibilities.

**Aims:**

This observational study aimed to investigate factors (sociodemographic characteristics of caregivers and mental health and/or addiction concerns of the caregiver and their youth) that predict perceived negative changes in caregiving responsibilities among adult caregivers (aged 18+ years) of children and youth (aged 0–25 years) in Ontario, Canada, during the COVID-19 pandemic.

**Method:**

Data were collected from 1381 caregivers of children and youth between January and March of 2022 through a representative cross-sectional survey completed online. Logistic regression was conducted to determine predictors contributing to perceived negative changes in caregiving responsibilities.

**Results:**

Among the sociodemographic characteristics, only ethnicity significantly predicted outcome. Higher caregiver strain (odds ratio [OR] = 10.567, 95% CI = 6.614–16.882, *P* < 0.001), worsened personal mental health (OR = 1.945, 95% CI = 1.474–2.567, *P* < 0.001), a greater number of children/youth cared for per caregiver (OR = 1.368, 95% CI = 1.180–1.587, *P* < 0.001), dissatisfaction with the availability of social supports (OR = 1.768, 95% CI = 1.297–2.409, *P* < 0.001) and negative changes in mental well-being in at least one child/youth (OR = 2.277, 95% CI = 1.660–3.123, *P* < 0.001) predicted negative changes in caregiving responsibilities.

**Conclusion:**

These results support further exploration of the implications of negative perceptions of caregiving responsibilities and what processes might be implemented to improve these perceptions and the outcomes.

The COVID-19 pandemic has caused long-lasting changes in many people's employment, social relationships, and mental and physical well-being. Caregivers of youth, children and seniors, representing 7.8 million Canadians (25% of the total Canadian population), tend to suffer in all aspects of life (finance, social, physical, emotional and mental) owing to their unique role in society during the pandemic.^1–3^ Caregivers are people who tend to someone else's needs for a period of time, and the context of this study refers to caregivers of children and youth.^4^ They act as a social support system, assist with daily tasks and ensure basic needs are met.^4^ Caregivers often face challenges and difficulties, including poor time management, worsening emotional and physical stress, lack of privacy, financial strain and sleep deprivation.^5–9^ Caregivers can struggle to balance this role with other responsibilities in their lives, and these strains can affect the youth they care for emotionally and mentally.^10^ Since the COVID-19 pandemic began, many challenges typically faced by caregivers have been exacerbated, leading to physical and mental deterioration of these caregivers.^10–12^ Children may have been at home for online schooling, routines may have been disrupted owing to limited access to childcare and social support systems, and many children and youth with mental health and/or addiction (MHA) concerns may have had diminished access to mental healthcare.^10^ The increased stress associated with the pandemic also led to increased alcohol use in youth during the pandemic.^13,14^ This has led to increased stress in youth and caregivers.

Recent studies have shown that COVID-19 has negatively affected caregivers of children and youth and has been associated with substantial changes in caregiving responsibilities.^15–20^ For example, when family caregivers were compared with non-caregivers in Pittsburgh, caregivers reported higher levels of anxiety and depression, increased food insecurity and financial worries, and lower social participation.^15^ Furthermore, those with more COVID-19-related disruptions had worsening mental health and physical outcomes, owing to the increased difficulties added to their responsibilities, and thus had more negative perceptions of their responsibilities.^15^ In Germany, the care situation of caregivers has worsened during the pandemic; 25.5% of caregivers that were unable to receive professional help reported that their responsibilities worsened during the pandemic, including overabundant demands, loss of social support and problems implementing COVID-19 measures.^16^ Caregivers had diminished abilities to complete their responsibilities owing to pandemic-induced changes in their responsibilities, and this had a substantial negative impact on their physical and mental health.^16^ In Japan, caregivers of children aged 3 to 14 years had an increase in mental distress compared with how they felt before the COVID-19 pandemic, with 24.1% reporting moderate mental distress and 29.3% reporting severe mental distress.^20^ It was also found that such mental distress could stem from changes in daily routine (increase in screen time, decrease in time spent outside, closure of schools) and increases in child health issues.^20^ Furthermore, caregivers’ stress during the COVID-19 pandemic has been shown to be internalised by children, causing psychological and behavioural issues including yelling/screaming, name-calling and blaming others.^20–22^ Thus, there is a need to continue to understand how the pandemic has affected the perception of caregiving responsibilities for children/youth over time in other jurisdictions, including Canada. As the pandemic has progressed, it has affected caregiving in an ever-changing way, as children and youth have returned to school and caregivers are going back to work and broader social contact. This current study aimed to investigate the sociodemographic and caregiver and child/youth mental health factors associated with perceived changes in caregiving responsibilities among caregivers of children and youth aged 0–25 years in Ontario, Canada, during the later stages of the COVID-19 pandemic.

## Method

### Study design and participants

The current study used wave 3 data from the COVID-19 Mental Health & Addictions Service Impacts & Care Needs Study, which aimed to examine the effects of the COVID-19 pandemic on mental health and substance use among adults residing in Ontario, Canada. Data reported in this study were collected from January to March 2022. See the study protocol for details regarding participant inclusion/exclusion criteria, recruitment and sampling.^23^ The authors assert that all procedures contributing to this work comply with the ethical standards of the relevant national and institutional committees on human experimentation and with the Helsinki Declaration of 1975, as revised in 2008. All procedures involving human subjects/patients were approved by Sunnybrook Health Sciences Centre Research Ethics Board (ref. 1931). Written informed consent was obtained from all participants.

Participants (*n* = 5000) were adults over the age of 18 living in Ontario, Canada. Data from 1381 participants who identified caregivers of children/youth aged 0–25 years were used for the purposes of this analysis.

### Measures

Variables related to the impact of the COVID-19 pandemic on caregiving responsibilities in relation to participants’ own and their child/youth's MHA concerns and sociodemographic characteristics were assessed through a series of measures. Complete details of the following measures can be found in the study protocol, including the sociodemographic characteristics of the overall study sample (*n* = 5000).^23^ Caregiver strain during the pandemic was assessed using the Peabody Treatment Progress Battery 2010 and the Caregiver Strain Questionnaire-Short Form 7 (Adult Caregiver) questionnaire (both Cronbach's α = 0.89).^24^ The total strain was further categorised into tertiles: low (score between 2 and 3.83), medium (3.83 to 7.0) and high (7.0 to 10).^24^ Depression and anxiety were assessed using the DSM-5 Self-Rated Level 1 Cross-Cutting Symptom Assessment for Adults (Cronbach's α = 0.81).^25^ Alcohol use was assessed using the Alcohol, Smoking and Substance Involvement Screening Test version 3.0 (Cronbach's α = 0.85).^26,27^

Six items about changes in caregiving responsibilities were created for the purpose of this study. Participants were asked if they had received any of a list of MHA services or supports in the past 3–4 months. Analysis was performed with a dichotomised response of yes (had access to at least one service/support) or no (received no service/support). Satisfaction with the availability of various types of social support (friends, family, romantic partners, community, co-workers, pets) since the COVID-19 pandemic was also assessed using a three-point Likert scale.^23^ Analysis was performed with a dichotomised response of yes (satisfied with the availability of social support) or no (neither satisfied nor dissatisfied or not satisfied with the availability of social support). Caregivers were also asked to estimate the degree to which their child/youth's mental well-being had changed from before the pandemic using a three-point Likert scale.^23^ Analysis was performed with a dichotomised response in at least one child/youth per caregiver of either a positive or no change as distinct from a negative change. Caregivers were asked whether or not at least one child/youth per caregiver had used at least one substance in an unhealthy/excessive manner since the pandemic.^23^ Analysis was performed with a dichotomised response of yes (used at least one substance in an excessive/unhealthy manner in at least one child/youth per caregiver) or no (had no excessive/unhealthy substance use in any child/youth cared for by caregiver). Access to MHA services since the pandemic commenced was also assessed through a dichotomised response of yes/no (yes: a service was accessed by at least one child/youth per caregiver; or no: no service was accessed by any youth). Participants were asked how their caregiving responsibilities had affected their well-being since the pandemic commenced.^23^ Analysis was performed with a dichotomised response of a positive or no impact as distinct from a negative impact.

### Statistical analysis

Data analysis was conducted in SPSS version 28.0 (IBM Corporation) with statistical significance set at *P* < 0.05 (two-tailed). Logistic regressions were performed with independent variables, namely sociodemographic characteristics, caregivers’ and children/youth's MHA concerns, and the dependent variables, namely, change in caregiving responsibilities. There was no evidence of multicollinearity among the independent variables (all variance inflation factor values < 2.5).

## Results

### General characteristics

Demographics for the sample are presented in Table 1. The age range for this sample of caregivers (*n* = 1381) was 18 to 91 years, with an average mean (±s.d.) of 43.60 years (±10.47) (Table 1). In this study, 649 (46.0%) identified as male, and 719 (52.1%) identified as female. The majority (480, 34.8%) lived in Central Ontario, were Caucasian (893, 64.7%), had at least some post-secondary education (1271, 92.0%) and were married/common-law partners (1185, 85.8%) (Table 1). These caregivers cared for a total of 2423 children/youth between the ages of 0 and 25 years with a mean (±s.d.) of 1.8 (±0.9) children/youth per caregiver (Table 1). The mean age of children/youth being cared for was 10.3 years (±7.0) (Table 2). Of the children and youth being cared for, there were 1284 (53.0%) males, 1129 (46.6%) females and ten (0.4%) who were reported to be non-binary (Table 2).

**Table 1:** Baseline characteristics of caregivers^a^

	Total (*n* = 1381)
Age group, years	
18–24	25 (1.8)
25–34	284 (20.6)
35–44	453 (32.8)
45–54	427 (30.9)
55–64	152 (11.0)
65–74	35 (2.5)
75–84	4 (0.3)
85–94	1 (0.1)
Gender	
Male	649 (47.0)
Female	719 (52.1)
Non-binary	13 (0.9)
Geographical region	
Toronto	169 (12.2)
Southwestern Ontario	340 (24.6)
Eastern Ontario	257 (18.6)
Central Ontario	480 (34.8)
Northern Ontario	135 (9.8)
Ethnicity	
Black	55 (4.0)
Caucasian	893 (64.7)
East Asian	151 (10.9)
Indigenous	19 (1.4)
Latino	18 (1.3)
Middle Eastern	26 (1.9)
Other	86 (6.2)
South Asian	104 (7.5)
Southeast Asian	29 (2.1)
Level of education	
High school or less	110 (8.0)
Some post-secondary or more	1271 (92.0)
Marital status	
Married/common-law	1185 (85.8)
Divorced/widowed/single	196 (14.2)
Number of children/youth per caregiver	
1	638 (46.2)
2	536 (38.8)
3	149 (10.8)
4	41 (3.0)
5	8 (0.6)
6	5 (0.4)
7	2 (0.1)
8	1 (0.1)
9	0 (0.0)
10	1 (0.1)

a. Reported as *n* (%).

**Table 2:** Baseline characteristics of children/youth^a^

	Total (*n* = 2423)
Age group, years	
0–4	590 (25.6)
5–9	457 (19.8)
10–14	534 (23.1)
15–19	459 (20.0)
20–25	266 (11.5)
Gender	
Male	1284 (53.0)
Female	1129 (46.6)
Non-binary	10 (0.4)

a. Reported as *n* (%).

### Impact of COVID-19 pandemic on caregivers

#### Caregiver strain

Caregivers scored a mean (±s.d.) of 2.22 ± 1.02 (range 1–5) on objective strain and a mean of 2.46 ± 1.12 (range 1–5) on subjective strain, and 4.68 ± 2.01 (range 2–10) on total caregiver strain (Table 3). Five hundred and forty-seven (39.6%) caregivers were in the low-strain group, 652 (47.2%) in the medium-strain group and 182 (13.2%) in the high-strain group (Table 3).

**Table 3:** Impacts of COVID-19 pandemic on caregivers

	Total (*n* = 1381)
Caregiver strain^a,b^	
Objective strain	2.22 ± 1.02
Subjective strain	2.46 ± 1.12
Total caregiver strain	4.68 ± 2.01
Changes to mental health^c^	
Worse	629 (45.5)
Unchanged	460 (33.3)
Better	292 (21.1)
Depression^c,d^	
Rating less than 2	720 (52.1)
Rating 2 or greater	661 (47.9)
Anxiety^c,d^	
Rating less than 2	668 (48.4)
Rating 2 or greater	713 (51.6)
Alcohol use risk levels^c,e^	
None	211 (15.3)
Low risk (0–10)	880 (63.7)
Moderate risk (11–26)	219 (15.9)
High risk (27+)	71 (5.1)
Access to MHA services or support^c^	
Yes	331 (24.0)
No	1050 (76.0)
Level of satisfaction with social support^c^	
Satisfied	1058 (76.6)
Dissatisfied	323 (23.4)
Caregiving responsibilities^c^	
Positive/neutral change	933 (67.6)
Negative change	448 (32.4)

MHA, mental health and/or addiction.

a. Reported as mean ± s.d.

b. Measured based on Peabody Treatment Progress Battery 2010 and the Caregiver Strain Questionnaire-Short Form 7 (Adult Caregiver) questionnaire.

c. Reported as *n* (%).

d. Measured based on DSM-5 Self-Rated Level 1 Cross-Cutting Symptom Measure–Adult.

e. Measured based on Alcohol, Smoking and Substance Involvement Screening Test.

#### Depression, anxiety and substance use

Since the pandemic was declared, almost half of the caregivers indicated that their mental health had worsened (*n* = 629, 45.5%) (Table 3). It was found that 211 (15.3%) were at no risk, 880 (63.7%) were at low risk, 219 (15.9%) were at moderate risk and 71 (5.1%) were at high risk (Table 3) for alcohol misuse.

#### Mental health and/or addictions services or support

Over three-quarters of caregivers did not receive MHA services or support (*n* = 1050, 76%). By contrast, 1048 (76.6%) were satisfied with their level of social support since the pandemic was declared (Table 3).

#### Caregiving responsibilities

Four hundred and forty-eight (32.4%) caregivers indicated that their caregiving responsibilities had had a negative impact on their well-being since the pandemic was declared (Table 3).

### Impact of COVID-19 pandemic on children/youth

A total of 981 (71.0%) caregivers reported negative mental well-being changes in at least one child/youth that they were caring for, and 243 (17.6%) indicated that at least one child/youth had signs of MHA issues since the pandemic began (Table 4). In addition, 181 (13.1%) caregivers had at least one child/youth with unhealthy/excessive substance use, and 157 (11.4%) caregivers had at least one child/youth accessing MHA services or support (Table 4).

**Table 4:** Caregiver's perception of impact of COVID-19 pandemic on children/youth^a^

	Total (*n* = 1381)
Changes in mental well-being^b^	
Yes	400 (29.0)
No	981 (71.0)
Signs of MHA issues^b^	
Yes	243 (17.6)
No	1138 (82.4)
Unhealthy/excessive substance use^b^	
Yes	181 (13.1)
No	1200 (86.9)
Access to MHA services or support^b^	
Yes	157 (11.4)
No	1224 (88.6)

MHA, mental health and/or addiction.

a. Reported as percentage (*n*).

b. In at least one child/youth per caregiver.

### Predictors of perceived changes in caregiving responsibilities

We used logistic regression to analyse the relationship between sociodemographic characteristics and caregivers’ and children/youth's MHA concerns (independent variables) and perceived changes in caregiving responsibilities (dependent variable) (Table 5). Of the sociodemographic characteristics, only ethnicity significantly predicted changes in caregiving responsibilities. The group including Black, Asian, Indigenous, Latino and Middle Eastern individuals had significantly lower odds of perceived negative changes in caregiving responsibilities compared with those identified as White (odds ratio [OR] = 0.691, 95% CI = 0.509–0.939, *P* = 0.018). For caregivers, higher caregiver strain (OR = 10.567, 95% CI = 6.614–16.882), worsened personal mental health (OR = 1.945, 95% CI = 1.474–2.567), a greater number of children/youth cared for per caregiver (OR = 1.368, 95% CI = 1.180–1.587) and dissatisfaction with the availability of social supports (OR = 1.768, 95% CI = 1.297–2.409, all *P* < 0.001) predicted negative changes in caregiving responsibilities. Depression (OR = 1.186, 95% CI = 0.845–1.664, *P* = 0.325); anxiety (OR = 0.729, 95% CI = 0.516–1.029, *P* = 0.072); low (OR = 1.306, 95% CI = 0.864–1.973, *P* = 0.206), moderate (OR = 0.879, 95% CI = 0.527–1.466, *P* = 0.621) and high (OR = 0.534, 95% CI = 0.259–2.103, *P* = 0.090) risk of alcohol misuse; and access to MHA services/support (OR = 1.136, 95% CI = 0.814–1.585, *P* = 0.453) did not predict negative changes in caregiving responsibilities. Negative changes in mental well-being (OR = 2.277, 95% CI = 1.660–3.123, *P* < 0.001) in at least one child/youth per caregiver significantly predicted perceived negative changes in caregiving responsibilities, whereas signs of MHA concerns (OR = 1.369, 95% CI = 0.842–2.227, *P* = 0.205), substance use (OR = 0.767, 95% CI = 0.478–1.138, *P* = 0.169) and access to MHA services/support (OR = 1.108, 95% CI = 0.628–1.955, *P* = 0.724) in at least one child/youth did not.

**Table 5:** Logistic regression for predictors of negative changes in caregiving responsibilities

Predictors	B	s.e.	OR	95% CI		Sig.
Demographics						
Non-White	−0.369	0.156	0.691	0.509	0.939	0.018^a^
Some postsecondary or more	0.507	0.273	1.661	0.972	2.838	0.064
Urban	0.122	0.230	1.129	0.720	1.771	0.596
Age	−0.014	0.008	0.986	0.972	1.001	0.070
Male	−0.239	0.142	0.787	0.596	1.039	0.92
Marital status	−0.383	0.207	0.682	0.455	1.022	0.064
Caregivers						
High caregiver strain^b^	2.358	0.239	10.567	6.614	16.882	<0.001^a^
Medium caregiver strain^b^	1.279	0.167	3.593	2.588	4.987	<0.001^a^
Worse mental health	0.665	0.142	1.945	1.474	2.567	<0.001^a^
Dissatisfied with social support	0.570	0.158	1.768	1.297	2.409	<0.001^a^
Number of Child/youth	0.313	0.076	1.368	1.180	1.587	<0.001^a^
Low alcohol use risk level^c^	0.267	0.211	1.306	0.864	1.973	0.206
Depression^d^	0.170	0.173	1.186	0.845	1.664	0.325
Access to MHA services	0.128	0.170	1.136	0.814	1.585	0.453
Medium alcohol use risk level^c^	−0.129	0.261	0.879	0.527	1.466	0.621
Anxiety^d^	−0.317	0.176	0.729	0.516	1.029	0.072
High alcohol use risk level^c^	−0.627	0.370	0.534	0.259	1.103	0.090
Caregiver perceptions of children/youth						
Changes in mental well-being^e^	0.823	0.161	2.277	1.660	3.123	<0.001^a^
Signs of MHA concerns^e^	0.314	0.248	1.369	0.842	2.227	0.205
Access to MHA services^e^	0.102	0.290	1.108	0.628	1.955	0.724
Unhealthy substance use^e^	-0.305	0.221	0.767	0.478	1.138	0.169

OR, odds ratio; MHA, mental health and/or addiction.

a. *P* < 0.05.

b. Measured based on Peabody Treatment Progress Battery 2010 and Caregiver Strain Questionnaire-Short Form 7 (Adult Caregiver) questionnaire.

c. Measured based on Alcohol, Smoking and Substance Involvement Screening Test.

d. Measured based on DSM-5 Self-Rated Level 1 Cross-Cutting Symptom Measure–Adult.

e. In at least one child/youth per caregiver.

## Discussion

The results of this study suggest that ethnicity, caregiver strain, negative changes in mental health for caregivers, negative changes in mental health for their children/youth, more children/youth cared by per caregiver and dissatisfaction with social supports were all independently associated with negative perceptions of changes in caregiving responsibilities. The COVID-19 pandemic resulted in new and increased responsibilities for caregivers of children and youth. Caregivers helped children and youth with online schooling by providing technology to access online platforms and necessities along with support in doing so, while simultaneously working from home.^5^ The burden of juggling multiple responsibilities while being isolated from social support may be associated with negative perceptions of caregiving responsibilities. Previous studies of the relationships between caregiver strain, mental health, number of children/youth, satisfaction with social support and caregiving responsibilities have reported similar results.^28,29^ A prior study considered adult caregivers (aged 18+ years) caring for children under the age of 18 years in the USA during the pandemic and showed that there were significant links between parents’ caregiving burden, mental health, and perceptions of children's stress, which were in turn significantly linked to child–parent closeness and conflict.^29^ Complementary results in another study of caregivers of at least one child between the ages of 6 and 18 years during the pandemic in the USA showed that having a set routine could buffer negatively perceived changes as a result of the pandemic.^28^ This shows that the caregivers were affected more by the idea of additional responsibilities than by the actual responsibility. Given these findings, further work is needed to explore how the severity of caregivers’ and youth's MHA concerns, caregiver strain and social support affect perceived caregivers’ responsibilities.

Compared with caregivers of underrepresented groups, White caregivers were more likely to report perceived negative changes in caregiving responsibilities. There may be several explanations for this finding. First, it is possible that cultural experiences may affect cognitive processes.^30^ Second, other variables in the model, such as caregiver strain, access to supports, social supports, etc., may have moderated the relationship between race and caregiving perceptions differently than expected. By contrast, a previous study, albeit focusing on caregivers caring for adult cancer patients during the pandemic, reported that caregivers of underrepresented groups were more likely to perceive higher negative perceptions in caregiving responsibilities.^31^ Future research should explore the relationship between caregiving responsibilities and ethnicity in more depth. This might include exploring protective and precipitating factors that may be different between groups. In addition, whereas this study found that gender did not significantly predict perceived negative changes in caregiving responsibilities, a prior study found that caregivers who were female had more strain and burden.^31^ Findings of this study suggest that many sociodemographic characteristics do not heavily influence perceived changes in caregiving responsibilities; these results are surprising in the context of the published work in the field. There could be different possible perceptions as to why there is a difference in findings, including differences in the sample population, survey service and percentage of females in the sample. The prior study looked at a sample population in the USA using Amazon Mechanical Turk with 42% females,^31^ whereas the present study was conducted in Canada through AskingCanadians with 52% females. Thus, future exploration is warranted to further understand the relationships between gender and caregiving roles.

Caregivers’ perception of changes in mental well-being in their children/youth significantly predicted perceived negative changes in caregiving responsibilities. Previous studies of the relationship between changes in mental health and caregiving responsibilities have shown similar results, despite investigating various populations in different regions.^29,32^ Caregiver burden, perceived child stress and conflict in child–parent relationships have all been found to be positively associated with one another in the USA.^29^ A prior study found that as a result of the pandemic, primary school children in Turkey developed sleeping problems, anger issues, fidgeting, restlessness, appetite problems, sadness, etc., as perceived by their caregivers.^32^ There was a significant association between these symptoms and perceived stress in caregivers. Children also seem to internalise the stress and burden faced by their caregivers, leading to the development of MHA signs.^19^

### Strengths and limitations

Participants in this study were a representative sample of residents in Ontario, improving the generalisability of the findings to the population. However, the study was conducted through an online survey, limiting the sample to those with internet access. Furthermore, the responses regarding children and youth's MHA concerns and access to MHA services and/or support were based on the caregivers’ perceptions, which might not have been accurate depictions; it is possible that children and youth may hide their symptoms or service involvement from their caregivers. Thus, it is also important to evaluate these factors with children and youth themselves directly wherever possible. In addition, caregivers were asked to compare their current caregiving responsibilities with those before the pandemic, which was over 2 years prior; this may have led to recall bias, affecting the validity of the data. It is also important to acknowledge that not all the changes in the lives of individual caregivers and their youths were solely the result of the pandemic; indeed, there may be many life events and intercurrent medical and social issues that affect the lives of caregivers, the measurement and the impact of which were beyond the scope of this study. Moreover, this study included an evaluation of a heterogeneous group of youth who were receiving care from caregivers. It is possible that certain subgroups (those with chronic illness, acute illness, physical disabilities, etc.) may have different levels of strain; it was beyond the scope of the current study to evaluate the differences between these different subgroups, but this may be a valuable issue to explore in future research. Last, the cross-sectional nature of this study limits the conclusions to relationships observed at one particular time point in the COVID-19 pandemic. Future studies could explore perceived negative changes in caregiving responsibilities as a result of the COVID-19 pandemic longitudinally to better determine how these relationships evolved over time (in caregivers and youth pairs).

### Future implications

This study examined the factors that contributed to perceived negative changes in caregiving responsibilities among adult caregivers (aged 18+ years) of children and youth (aged 0–25 years) in Ontario, Canada, during the COVID-19 pandemic. The findings show that certain factors (caregiver strain, negative changes in mental health for caregivers and their children/youth, high number of children/youth cared for per caregiver and dissatisfaction with social supports) were predictors of negatively perceived changes in caregiving responsibilities. Other factors including screening for risk of depression and anxiety, access to MHA services/supports and signs of MHA concerns in children/youth were not associated with the outcome. Ethnicity was the only sociodemographic factor that significantly predicted the outcome. These findings could lead to a greater understanding of the impact the pandemic has had on caregiving responsibilities and how this has affected different populations across Ontario. They may also help to inform healthcare providers who seek to support caregivers of children and youth by developing their understanding of the nature of the challenges experienced during the pandemic.

## Data Availability

Requests for access to deidentified data should be directed to the corresponding author (anthony.levitt@sunnybrook.ca). Data may be shared upon reasonable request and pending ethics approval.

## References

[1] Arriagada P. The Experiences and Needs of Older Caregivers in Canada. Statistics Canada, 2020 (https://www150.statcan.gc.ca/n1/pub/75-006-x/2020001/article/00007-eng.htm [cited 4 Jul 2022]).

[2] Centers for Disease Control and Prevention. For Caregivers, Family, and Friends. CDC, 2022 (https://www.cdc.gov/aging/caregiving/index.htm [cited 4 Jul 2022]).

[3] Centers for Disease Control and Prevention. Women, Caregiving, and COVID-19. CDC, 2021 (https://www.cdc.gov/women/caregivers-covid-19/index.html [cited 4 Jul 2022]).

[4] McQuay J. What Is a Caregiver? Johns Hopkins Bayview Medical Center, 2020 (https://www.hopkinsmedicine.org/about/community_health/johns-hopkins-bayview/services/called_to_care/what_is_a_caregiver.html [cited 4 Jul 2022]).

[5] Oruche UM, Draucker CB, Al-Khattab H, Cravens HA, Lowry B, Lindsey LM. The challenges for primary caregivers of adolescents with disruptive behavior disorders. J Fam Nurs 2014; 21(1): 149–67. 25504213 10.1177/1074840714562027 PMC4575286

[6] Kostopoulos E, Sinopidis X, Gidaris D, Karantaglis N, Cassimos D, Gkentzi D, et al. Parents under siege: the psychological impact of COVID-19 outbreak on children's caregivers. Swiss Med Weekly 2021; 151(3132): w30012. 10.4414/SMW.2021.w30012 34407364

[7] Dionne-Odom JN, Azuero A, Taylor RA, Wells RD, Hendricks BA, Bechthold AC, et al. Resilience, preparedness, and distress among family caregivers of patients with advanced cancer. Support Care Cancer 2021; 29(11): 6913–20. 34031751 10.1007/s00520-021-06265-y PMC9733586

[8] Kazemi A, Azimian J, Mafi M, Allen KA, Motalebi SA. Caregiver burden and coping strategies in caregivers of older patients with stroke. BMC Psychol 2021; 9(1): 51. 33794995 10.1186/s40359-021-00556-z PMC8017750

[9] Bradshaw J, Gillespie S, McCracken C, King BH, McCracken JT, Johnson CR, et al. Predictors of caregiver strain for parents of children with autism spectrum disorder. J Autism Dev Disord 2021; 51(9): 3039–49. 33151499 10.1007/s10803-020-04625-x PMC10860166

[10] Gallagher-Mackay K, Srivastava P, Underwood K, Dhuey E, McCready L, Born K, et al. COVID-19 and education disruption in Ontario, emerging evidence on impacts. Science briefs of the Ontario COVID-19. Sci Advisory Table 2021; 2(34): 1–36.

[11] Landi G, Pakenham KI, Cattivelli R, Grandi S, Tossani E. Caregiving responsibilities and mental health outcomes in young adult carers during the COVID-19 pandemic: a longitudinal study. Int J Environ Res Public Health 2022; 19(22): 15149. 36429866 10.3390/ijerph192215149 PMC9690746

[12] Dhiman S, Shau PK, Reed WR, Ganesh GS, Goyal RK, Jain S. Impact of COVID-19 outbreak on mental health and perceived strain among caregivers tending children with special needs. Res Dev Disabil 2020; 107: 103790. 33091712 10.1016/j.ridd.2020.103790 PMC7538124

[13] Hartney E. The shadow pandemic of alcohol use during COVID-19: a Canadian health leadership imperative. Healthc Policy 2021; 16(4): 17–24. 34129475 10.12927/hcpol.2021.26502 PMC8200837

[14] Vanderbruggen N, Matthys F, Van Laere S, Zeeuws D, Santermans L, Van den Ameele S, et al. Self-reported alcohol, tobacco, and cannabis use during COVID-19 lockdown measures: results from a web-based survey. Eur Addict Res 2020; 26(6): 309–15. 32961535 10.1159/000510822 PMC7573904

[15] Beach SR, Schulz R, Donovan H, Rosland AM. Family caregiving during the COVID-19 pandemic. Gerontologist 2021; 61(5): 650–60. 33847355 10.1093/geront/gnab049 PMC8083337

[16] Budnick A, Hering C, Eggert S, Teubner C, Suhr R, Kuhlmey A, et al. Informal caregivers during the COVID-19 pandemic perceive additional burden: findings from an ad-hoc survey in Germany. BMC Health Serv Res 2021; 21(1): 353. 33863337 10.1186/s12913-021-06359-7 PMC8050992

[17] Pecor KW, Barbayannis G, Yang M, Johnson J, Materasso S, Borda M, et al. Quality of life changes during the COVID-19 pandemic for caregivers of children with ADHD and/or ASD. Int J Environ Res Public Health 2021; 18(7): 3667. 33915884 10.3390/ijerph18073667 PMC8037979

[18] Raviv T, Warren CM, Washburn JJ, Kanaley MK, Eihentale L, Goldenthal HJ, et al. Caregiver perceptions of children's psychological well-being during the COVID-19 pandemic. JAMA Netw Open 2021; 4(4): e2111103. 33914046 10.1001/jamanetworkopen.2021.11103 PMC8085728

[19] He Y, Ortiz R, Kishton R, Wood J, Fingerman M, Jacobs L, et al. In their own words: child and adolescent perceptions of caregiver stress during early COVID-19. Child Abuse Negl 2022; 124: 105452. 34954423 10.1016/j.chiabu.2021.105452 PMC8692067

[20] Horiuchi S, Shinohara R, Otawa S, Akiyama Y, Ooka T, Kojima R, et al. Caregivers’ mental distress and child health during the COVID-19 outbreak in Japan. PLoS One 2020; 15(12): e0243702. 33301517 10.1371/journal.pone.0243702 PMC7728265

[21] Tang S, Xiang M, Cheung T, Xiang YT. Mental health and its correlates among children and adolescents during COVID-19 school closure: the importance of parent-child discussion. J Affect Disord 2021; 279: 353–60. 33099049 10.1016/j.jad.2020.10.016 PMC7550131

[22] Vaughan EL, Feinn R, Bernard S, Brereton M, Kaufman J. Relationships between child emotional and behavioral symptoms and caregiver strain and parenting stress. J Fam Issues 2013; 34(4): 534–56. 24707069 10.1177/0192513X12440949 PMC3975620

[23] Markoulakis R, Khalid M, Da Silva A, Kodeeswaran S, Sinyor M, Cheung A, et al. Cross-sectional survey of the mental health and addictions effects, service impacts and care needs of children, youth and families during the COVID-19 pandemic: the COVID-19 MASC study protocol. BMJ Open 2022; 12: e066190. 10.1136/bmjopen-2022-066190 PMC9615177 36288837

[24] Bickman L, Athay MM, Riemer M, Lambert EW, Kelley SD, Breda C, et al. Manual of the Peabody Treatment Progress Battery. Vanderbilt University, 2010.

[25] Narrow WE, Clarke DE, Kuramoto SJ, Kraemer H, Kupfer D, Greiner L, et al. DSM-5 field trials in the United States and Canada, part III: development and reliability testing of a cross-cutting symptoms assessment for DSM-5. Am J Psychiatry 2013; 170(1): 71–82. 23111499 10.1176/appi.ajp.2012.12071000

[26] WHO ASSIST Working Group. The alcohol, smoking and substance involvement screening test (ASSIST): development, reliability, and feasibility. Addiction 2002; 97(9): 1183–94. 12199834 10.1046/j.1360-0443.2002.00185.x

[27] Humeniuk R, Dennington V, Ali R. *The Effectiveness of a Brief Intervention for Illicit Drugs Linked to the Alcohol, Smoking and Substance Involvement Screening Test (ASSIST) in Primary Health Care Settings: A Technical Report of Phase III Findings of the WHO ASSIST Randomized Controlled Trial*. WHO, 2008.

[28] Bates CR, Nicholson LM, Rea EM, Hagy HA, Bohnert A. Life interrupted: family routines buffer stress during the COVID-19 pandemic. J Child Fam Stud 2021; 30(11): 2641–51. 34404970 10.1007/s10826-021-02063-6 PMC8360776

[29] Russell BS, Hutchinson M, Tambling R, Tomkunas AJ, Horton AL. Initial challenges of caregiving during COVID-19: caregiver burden, mental health, and the parent-child relationship. Child Psychiatry Hum Dev 2020; 51(5): 671–82. 32749568 10.1007/s10578-020-01037-x PMC7398861

[30] Köster M, Yovsi R, Kärtner J. Cross-cultural differences in the generation of novel ideas in middle childhood. Front Psychol 2020; 11(1829): 118–25. 32903850 10.3389/fpsyg.2020.01829 PMC7438980

[31] Longacre ML, Miller MF, Fang CY. Racial and ethnic variations in caregiving-related physical, emotional, and financial strain during COVID-19 among those caring for adult cancer patients. Support Care Cancer 2021; 29(7): 4137–46. 33404809 10.1007/s00520-020-05933-9 PMC7785926

[32] Büber A, Aktaş Terzioğlu M. Caregiver's reports of their children's psychological symptoms after the start of the COVID-19 pandemic and caregiver's perceived stress in Turkey. Nordic J Psychiatry 2022; 76(3): 215–24. 10.1080/08039488.2021.1949492 34289778

